# Consumption of Alcoholic Beverages and Liquor Consumption by Michigan High School Students, 2011

**DOI:** 10.5888/pcd12.150290

**Published:** 2015-11-12

**Authors:** Katherine R. Gonzales, Thomas W. Largo, Corinne Miller, Dafna Kanny, Robert D. Brewer

**Affiliations:** Author Affiliations: Katherine R. Gonzales, Thomas W. Largo, Corinne Miller, Bureau of Disease Control, Prevention and Epidemiology, Michigan Department of Health and Human Services, Lansing, Michigan; Robert D. Brewer, Alcohol Program, Epidemiology and Surveillance Branch, Division of Population Health, National Center for Chronic Disease Prevention and Health Promotion, Centers for Disease Control and Prevention, Atlanta, Georgia.

## Abstract

**Introduction:**

Excessive alcohol consumption was responsible for approximately 4,300 annual deaths in the United States among people younger than 21 from 2006 through 2010. Underage drinking cost the United States $24.6 billion in 2006. Previous studies have shown that liquor is the most common type of alcohol consumed by high school students. However, little is known about the types of liquor consumed by youth or about the mixing of alcohol with energy drinks.

**Methods:**

The 2011 Michigan Youth Tobacco Survey was used to assess usual alcohol beverage consumption and liquor consumption and the mixing of alcohol with energy drinks by Michigan high school students. Beverage preferences were analyzed by demographic characteristics and drinking patterns.

**Results:**

Overall, 34.2% of Michigan high school students consumed alcohol in the past month, and 20.8% reported binge drinking. Among current drinkers, liquor was the most common type of alcohol consumed (51.2%), and vodka was the most prevalent type of liquor consumed by those who drank liquor (53.0%). The prevalence of liquor consumption was similar among binge drinkers and nonbinge drinkers, but binge drinkers who drank liquor were significantly more likely than nonbinge drinkers to consume vodka and to mix alcohol with energy drinks (49.0% vs 18.2%, respectively).

**Conclusions:**

Liquor is the most common type of alcoholic beverage consumed by Michigan high school students; vodka is the most common type of liquor consumed. Mixing alcohol and energy drinks is common, particularly among binge drinkers. *Community Guide* strategies for reducing excessive drinking (eg, increasing alcohol taxes) can reduce underage drinking.

## Introduction

Annually from 2006 through 2010, excessive alcohol consumption was responsible for approximately 4,300 deaths and 260,000 years of potential life lost in the United States among people younger than 21 years (1). In 2006, underage drinking cost the United States approximately $24.6 billion (2). More than 90% of the alcohol consumed by underage drinkers occurs during binge drinking episodes (3). Underage drinking is also associated with many health and social consequences, such as poor academic performance, interpersonal violence, injuries, risky sexual behavior, and unplanned pregnancies (4–6). Alcohol consumption by youth is also strongly correlated with alcohol consumption by adults at the state level (7), and youth often obtain the alcohol they consume from adults (8).

Recent studies identified liquor (eg, vodka, rum, scotch, bourbon, whiskey) as the usual type of alcohol consumed by high school students (9–13). Students who reported usually consuming liquor were also more likely to report frequent alcohol consumption and binge drinking than students who reported consuming other types of alcoholic beverages (11). However, limited information is available on the type of liquor that is consumed by underage youth and whether specific types of alcohol are more likely to be mixed with other types of beverages, such as energy drinks (eg, Monster, Red Bull). Understanding the type of alcohol usually consumed by youth is important for developing and targeting interventions to prevent underage and binge drinking, because alcohol control policies (eg, alcohol taxes) and alcohol advertising vary by beverage type (9,10). In addition, previous studies suggested that youth who mix alcohol with energy drinks are more likely to binge drink, consume more total drinks, have higher levels of alcohol intoxication, and experience alcohol-attributable harms (eg, sexual assault) than those who do not mix energy drinks with alcohol (14,15).

As part of its efforts to reduce underage drinking, the Michigan Department of Health and Human Services added supplemental questions on alcohol to its 2011 Youth Tobacco Survey. The purpose of this study was to use these survey findings to assess the type of alcoholic beverage (eg, beer, wine, liquor) usually consumed by Michigan high school students, the type of liquor consumed by students who drank liquor, the prevalence of mixing alcohol with energy drinks, and how these drinking behaviors varied by the demographic characteristics and drinking patterns (eg, binge drinking) of these students.

## Methods

The Michigan Youth Tobacco Survey (Michigan YTS) is a school-based survey of a random sample of public-school students in grades 9 through 12. Students surveyed complete an anonymous, self-administered questionnaire that consists of questions on tobacco use and on various environmental factors related to tobacco use, such as access to tobacco products in retail settings. In 2011, 4,142 Michigan high school students from 42 schools completed the Michigan YTS. The school response rate was 68%, the student response rate was 93%, and the overall response rate was 63%. Data were weighted by school, student nonresponse, and selected demographic characteristics (ie, the sex, race/ethnicity, and grade of students). Because the Michigan YTS is an established public health surveillance system, it does not require review of the Michigan Department of Health and Human Services Institutional Review Board.

To assess beverage-specific alcohol consumption by youth, 11 state-added questions on alcohol consumption were included in the 2011 Michigan YTS. These questions were developed in collaboration with the Excessive Alcohol Use Prevention Program of the National Center for Chronic Disease Prevention and Health Promotion at the Centers for Disease Control and Prevention, the Center on Alcohol Marketing and Youth at the Johns Hopkins Bloomberg School of Public Health, the Michigan Liquor Control Commission, and other experts in alcohol and public health. The questions added were based on questions that were used in other surveys, such as the Youth Risk Behavior Survey and a panel survey of beverage-specific alcohol consumption (12). Students were asked about their 30-day consumption of alcohol; binge drinking; the largest number of drinks consumed on any occasion; the usual type of alcohol consumed (ie, students could select one alcoholic beverage type that they usually consumed in the past 30 days); the usual type of liquor consumed; the usual brand of liquor, beer, or flavored alcoholic beverage consumed; how frequently they mixed alcohol with energy drinks; their usual drinking location; and various direct consequences that could have resulted from alcohol consumption (eg, getting into a fight, riding with a driver who had been drinking).

Current drinkers were defined as students who reported consuming at least 1 alcoholic drink in the 30 days before survey administration. Binge drinkers were defined as current drinkers who reported consuming 5 or more alcoholic drinks in a row (ie, within a couple of hours) during the 30 days before survey administration. Nonbinge drinkers were students who identified themselves as current drinkers but did not report consuming 5 or more alcoholic drinks in a row during the past 30 days.

Analyses were conducted using SAS, version 9.2 (SAS Institute, Inc) to account for the complex survey design and to produce estimates that were representative of Michigan’s high school student population. Prevalence estimates with 95% confidence intervals were calculated. Pearson’s χ^2^was used to test for differences in the prevalence of beverage-specific alcohol consumption between groups defined by various demographic factors (ie, sex, grade, and race/ethnicity) and by drinking patterns (eg, drinking frequency, drinking intensity, usual place of consumption, usual alcohol type consumed, usual liquor type consumed, mixing alcohol and energy drinks).

## Results

Overall, 34.2% of Michigan high school students reported current alcohol use; 20.8% of students were binge drinkers (Table 1). Binge drinking prevalence was higher in each successive grade, ranging from 14.3% among 9th-grade students to 35.9% among 12th-grade students. However, there were no significant differences in binge drinking prevalence by sex or by race/ethnicity.

**Table 1:** Prevalence of Binge, Nonbinge, or Nondrinking Among Michigan 9th Through 12th Grade Students, by Demographic Characteristics, Michigan Youth Tobacco Survey, 2011

Demographic Characteristic	Binge Drinkers^a^, % (95% CI)	Nonbinge Drinkers^b^, % (95% CI)	Nondrinkers^c^, % (95% CI)
**Overall** (n = 3,985)	20.8 (17.5–24.1)	13.4 (11.9–15.0)	65.8 (62.1–69.4)
**Sex**			
Female (n = 2,009)	19.4 (16.2–22.7)	15.0 (13.2–16.9)	65.5 (61.8–69.3)
Male (n = 1,947)	22.2 (17.9–26.5)	11.8 (9.6–14.0)	66.0 (61.1–70.9)
**Grade**			
9th (n = 1,612)	14.3 (10.9–7.7)	11.9 (9.7–14.2)	73.8 (69.5–78.1)
10th (n = 1,308)	16.2 (12.8–19.6)	12.9 (10.8–14.9)	71.0 (66.7–75.2)
11th (n = 663)	18.7 (14.3–23.2)	14.4 (10.8–18.0)	66.9 (61.5–72.2)
12th (n = 394)	35.9 (27.7–44.0)	14.8 (11.2–18.4)	49.3 (39.7–58.9)
**Race/ethnicity**			
White (n = 2,988)	21.3 (17.2–25.4)	13.4 (11.8–15.1)	65.2 (60.5–70.0)
Black (n = 478)	18.4 (11.4–25.4)	15.5 (11.0–20.0)	66.1 (58.7–73.5)
Hispanic (n = 182)	24.2 (15.6–32.8)	14.6 (9.0–20.3)	61.1 (52.9–69.4)
Other (n = 285)	18.4 (10.5–26.2)	6.2 (4.2–8.3)	75.4 (67.3–83.5)

Abbreviation: CI, confidence interval.

a Binge drinkers were defined as students who had 5 or more drinks of alcohol in a row within a couple of hours on at least 1 day during the 30 days before the survey.

b Nonbinge drinkers were defined as students who had at least 1 drink of alcohol on at least 1 day during the 30 days before the survey who did not report binge drinking.

c Nondrinkers were defined as students who reported consuming no alcoholic drinks in the 30 days before the survey.

Among students who reported drinking in the past 30 days, binge drinkers had a significantly higher prevalence of drinking in someone else’s home than did nonbinge drinkers (61.6% vs 44.0%;* P* < .001) and had more than 5 times the prevalence of drinking on 10 or more days in the past 30 days than nonbinge drinkers (24.4% vs 4.5%;* P* < .001) (Table 2). Binge drinkers also had more than twice the prevalence of mixing energy drinks with alcohol compared with nonbinge drinkers (49.0% vs 18.2%; *P* < .001).

**Table 2:** Distribution of Alcohol Consumption Characteristics Among Students Who Were Binge or Nonbinge Drinkers, Michigan Youth Tobacco Survey, 2011

Characteristic	Binge Drinkers^a^, n = 698, % (95% CI)	Nonbinge Drinkers^b^, n = 504, % (95% CI)
**Usual place of consumption**		
My home (n = 307)	22.7 (18.0–27.5)	37.7 (32.2–43.2)
Someone else’s home (n = 529)	61.6 (55.4–67.7)	44.0 (37.6–50.4)
Restaurant/bar (n = 30)	3.6 (1.9–5.3)	2.7 (0.6–4.9)
Other place^c^ (n = 123)	12.1 (8.5–15.7)	15.6 (10.3–20.8)
**Number of drinking days**		
1 or 2 (n = 577)	27.3 (24.0–30.6)	75.3 (71.2–79.3)
3–9 (n = 434)	48.3 (44.2–52.4)	20.3 (16.4–24.1)
10–30 (n = 191)	24.4 (21.1–27.7)	4.5 (1.9–7.0)
**Mixed energy drinks and alcohol** (n = 431)	49.0 (43.7–54.2)	18.2 (13.1–23.3)

Abbreviation: CI, confidence interval.

a Binge drinkers were defined as students who had 5 or more drinks of alcohol in a row within a couple of hours on at least 1 day during the 30 days before the survey.

b Nonbinge drinkers were defined as students who had at least 1 drink of alcohol on at least 1 day during the 30 days before the survey who did not report binge drinking.

c Other place was defined as riding or driving in a car; at a beach, park, concert, or sporting event; or on school property.

Most Michigan high school students who drank alcohol reported usually consuming liquor (51.2%) (Table 3). The prevalence of liquor consumption was slightly higher for binge drinkers (54.2%) than for nonbinge drinkers (46.9%). Liquor was the most frequently reported usual type of alcohol consumed by current drinkers across all demographic groups and drinking patterns and was reported by most students who reported mixing alcohol and energy drinks (52.7%). Girls reported a significantly higher prevalence of drinking flavored alcoholic beverages than boys (21.0% vs 8.1%;* P* < .001), whereas boys reported a significantly higher prevalence of drinking beer than girls (24.3% vs 11.1%;* P* < .001). Other types of alcohol (wine and cordials) accounted for a small proportion (11.2%) of the usual type of alcohol consumed by Michigan high school students. Only 5.2% of current drinkers reported not having a usual beverage type.

**Table 3:** Distribution of Usual Types of Alcohol Consumed^a^ Among Current (Binge and Nonbinge) Drinkers, by Sociodemographic Characteristics and Consumption Patterns, Michigan Youth Tobacco Survey, 2011

Characteristic	Liquor, % (95% CI)	Beer, % (95% CI)	Flavored Alcoholic Beverages, % (95% CI)	No Usual Type, % (95% CI)	Other Type^b^, % (95% CI)
**Overall (n = 963)**	51.2 (47.5–54.8)	17.9 (14.4–21.5)	14.5 (11.0–18.1)	5.2 (3.2–7.3)	11.2 (7.0–15.3)
**Sex**					
Female (n = 485)	53.6 (46.2–61.0)	11.1 (7.2–15.1)	21.0 (15.3–26.6)	5.8 (2.6–8.9)	8.5 (3.9–13.1)
Male (n = 473)	49.0 (45.4–52.6)	24.3 (18.6–29.9)	8.1 (4.8–11.5)	4.7 (2.3–7.1)	13.9 (8.7–19.0)
**Grade**					
9th (n = 324)	51.4 (44.6–58.1)	16.2 (10.7–21.8)	15.1 (11.0–19.2)	6.0 (3.3–8.6)	11.4 (7.1–15.6)
10th (n = 294)	53.7 (47.6–59.7)	13.5 (9.6–17.5)	16.5 (10.9–22.2)	7.1 (4.0–10.1)	9.2 (5.5–13.0)
11th (n = 174)	49.9 (42.5–57.3)	13.4 (7.3–19.5)	18.6 (11.0–26.3)	2.4 (0.0–4.7)	15.7 (5.5–25.9)
12th (n = 170)	50.5 (41.6–59.4)	24.6 (18.6–30.6)	10.3 (4.5–16.1)	5.6 (0.8–10.4)	9.0 (4.1–13.9)
**Race/ethnicity**					
White (n = 746)	52.1 (48.5–55.7)	19.6 (15.3–23.9)	12.8 (9.0–16.7)	5.8 (3.7–7.9)	9.7 (5.5–13.8)
Black (n = 110)	48.9 (35.7–62.0)	10.1 (1.9–18.3)	23.4 (10.9–35.9)	3.1 (0.0–8.2)	14.5 (6.1–22.9)
Hispanic (n = 50)	55.1 (36.8–73.4)	16.0 (1.4–30.7)	11.7 (2.4–21.0)	4.0 (0.0–9.1)	13.1 (0.1–26.2)
**Drinking status**					
Nonbinge drinkers^c^ (n = 406)	46.9 (39.7–54.1)	17.1 (13.2–20.9)	17.6 (12.4–22.9)	5.5 (1.5–9.6)	12.9 (5.3–20.5)
Binge drinkers^d^ (n = 551)	54.2 (49.1–59.3)	18.6 (13.5–23.7)	12.1 (8.2–16.1)	5.1 (2.7–7.4)	10.0 (6.4–13.7)
**Usual place of consumption**					
My home (n = 340)	37.9 (30.5–45.3)	21.2 (15.5–26.9)	18.3 (12.6–24.0)	6.8 (3.9–9.8)	15.7 (9.2–22.2)
Someone else’s home (n = 546)	60.2 (54.7–65.7)	15.4 (9.8–21.1)	13.4 (8.0–18.7)	5.0 (3.3–6.6)	6.0 (2.8–9.2)
Other place^e^ (n = 163)	41.7 (31.6–51.7)	20.3 (12.7–27.9)	13.1 (6.0–20.2)	6.5 (0.3–12.7)	18.4 (11.9–24.9)
**Number of drinking days**					
1 or 2 (n = 463)	47.8 (41.5–54.1)	17.8 (13.6–22.0)	18.1 (12.5–23.7)	5.0 (1.6–8.4)	11.4 (5.1–17.7)
3–9 (n = 355)	57.6 (51.8–63.5)	16.2 (10.7–21.6)	11.7 (7.8–15.5)	4.9 (2.8–6.9)	9.7 (5.4–13.9)
10–30 (n = 145)	45.9 (36.6–55.2)	22.6 (14.9–30.4)	10.7 (2.4–19.0)	6.7 (0.5–13.0)	14.0 (6.8–21.3)
**Largest number of drinks on any occasion**					
1–3 (n = 474)	46.2 (41.0–51.3)	16.7 (11.9–21.5)	18.7 (14.2–23.1)	4.8 (1.3–8.3)	13.7 (7.7–19.7)
4 or 5 (n = 176)	56.9 (48.1–65.7)	12.3 (5.5–19.1)	18.3 (11.3–25.4)	5.2 (2.4–8.0)	7.3 (2.4–12.2)
≥6 (n = 328)	55.1 (49.9–60.3)	23.4 (17.0–29.8)	7.1 (4.0–10.3)	5.2 (1.3–9.1)	9.1 (5.5–12.8)
**Mixed energy drinks and alcohol** (n = 432)	52.7 (45.1–60.3)	18.0 (12.1–23.9)	12.2 (7.0–17.4)	5.2 (2.8–7.6)	12.0 (6.7–17.2)

Abbreviation: CI, confidence interval.

a Survey question to assess usual types of alcohol was, “During the past 30 days, what type of alcohol did you usually drink? (Select only one response)”.

b Other type of alcohol includes wine and cordials.

c Nonbinge drinkers were defined as students who had at least 1 drink of alcohol on at least 1 day during the 30 days before the survey, but did not report binge drinking.

d Binge drinkers were defined as students who had 5 or more drinks of alcohol in a row within a couple of hours on at least 1 day during the 30 days before the survey.

e Other place was defined as a bar or restaurant; riding or driving in a car; at a park, beach, concert, or sporting event; or on school property.

Vodka consumption was reported by 37.3% of current drinkers, making it by far the most common type of liquor consumed by Michigan high school students (Table 4). Vodka consumption was more common among binge drinkers (42.1%) than among nonbinge drinkers (30.7%) and was reported by almost half of the students who reported mixing alcohol and energy drinks. Vodka was the most common type of liquor consumed by current drinkers across all demographic groups, but it was somewhat more common among girls (42.8%) than boys (31.7%). Students who drank in someone else’s home also had a significantly higher prevalence of vodka consumption (49.7%) than those who usually drank in their own home (30.9%) or at some public place (30.9%). However, the prevalence of vodka consumption by current drinkers did not vary significantly by drinking frequency (the number of drinking days) or intensity (the largest number of drinks consumed on 1 occasion). Rum consumption was somewhat more common among boys than girls (15.4% vs 7.8%), as was the consumption of whiskey (13.0% vs 4.4%,). However, the prevalence of tequila consumption did not vary significantly by sex.

**Table 4:** Distribution of Usual Type of Liquor Consumed^a^ Among Current (Binge and Nonbinge) Drinkers, by Demographic Characteristics and Consumption Patterns, Michigan Youth Tobacco Survey, 2011

Characteristic	Didn’t Drink Liquor, % (95% CI)	Vodka, % (95% CI)	Rum, % (95% CI)	Whiskey, % (95% CI)	Tequila, % (95% CI)	No Usual Type, % (95% CI)	Other Type, % (95% CI)
**Overall** (n = 1,146)	19.1 (15.6–22.5)	37.3 (33.2–41.1)	11.6 (8.9–14.2)	8.7 (5.6–11.7)	4.6 (3.0–6.3)	10.1 (7.8–12.5)	8.6 (6.0–11.2)
**Sex**							
Female (n = 577)	16.7 (11.4–22.0)	42.8 (36.4–49.1)	7.8 (5.5–10.1)	4.4 (1.8–7.0)	5.8 (3.6–8.0)	13.0 (8.9–17.0)	9.6 (5.4–13.8)
Male (n = 561)	21.4 (18.6–24.3)	31.7 (28.2–35.2)	15.4 (11.5–19.4)	13.0 (9.3–16.8)	3.2 (1.2–5.2)	7.4 (4.1–10.6)	7.8 (4.8–10.8)
**Grade**							
9th (n = 384)	21.1 (13.9–28.3)	37.0 (30.9–43.2)	9.7 (5.8–13.6)	9.4 (5.8–13.1)	4.4 (2.4–6.4)	8.0 (4.2–11.9)	10.4 (6.5–14.2)
10th (n = 359)	20.6 (13.4–27.8)	33.5 (26.5–40.4)	9.1 (5.6–12.6)	8.2 (4.3–12.1)	4.1 (2.0–6.3)	11.6 (7.7–15.5)	12.9 (8.0–17.8)
11th (n = 212)	23.6 (16.4–30.8)	36.8 (27.6–45.9)	10.9 (6.5–15.4)	5.7 (3.1–8.4)	3.6 (1.3–5.9)	10.0 (4.7–15.3)	9.4 (3.9–15.0)
12th (n = 190)	13.7 (7.2–20.2)	40.4 (31.1–49.6)	14.8 (10.5–19.1)	10.6 (3.9–17.3)	5.7 (1.8–9.6)	10.6 (5.8–15.5)	4.2 (1.3–7.1)
**Race/ethnicity**							
White (n = 871)	19.9 (15.6–24.1)	39.4 (34.2–44.5)	12.3 (9.7–15.0)	8.9 (5.5–12.4)	3.7 (1.8–5.6)	9.7 (7.1–12.4)	6.0 (4.4–7.6)
Black (n = 141)	16.5 (9.2–23.9)	32.9 (21.4–44.5)	7.3 (1.2–13.3)	5.1 (1.3–8.9)	5.8 (0.5–11.1)	12.6 (3.6–21.5)	19.8 (11.0–28.6)
Hispanic (n = 63)	15.2 (7.4–23.0)	29.8 (16.5–43.1)	12.0 (2.5–21.4)	10.5 (1.4–19.7)	18.5 (2.2–34.9)	7.9 (2.0–13.9)	6.1 (0.1–12.0)
**Drinking behavior**							
Nonbinge drinkers^b^ (n = 491)	26.2 (22.3–30.0)	30.7 (23.5–38.0)	10.0 (5.9–14.2)	6.1 (3.3–8.9)	5.0 (2.3–7.7)	11.5 (7.7–15.3)	10.5 (6.4–14.6)
Binge drinkers^c^ (n = 647)	14.1 (10.0–18.2)	42.1 (38.2–46.1)	12.6 (9.3–15.9)	10.5 (6.8–14.3)	4.4 (2.0–6.8)	9.3 (6.5–12.1)	6.9 (4.3–9.5)
**Usual place of consumption**							
My home (n = 304)	14.2 (8.4–20.0)	30.9 (22.6–39.2)	10.5 (6.8–14.2)	10.9 (5.4–16.4)	7.6 (4.2–11.0)	14.3 (8.4–20.3)	11.6 (5.7–17.5)
Someone else’s home (n = 513)	8.3 (5.2–11.5)	49.7 (43.8–55.7)	12.7 (8.8–16.5)	7.9 (4.8–11.0)	3.0 (1.1–4.8)	10.1 (7.0–13.3)	8.2 (4.9–11.6)
Other place^d^ (n = 144)	13.2 (8.3–18.0)	30.9 (23.2–38.7)	20.0 (11.6–28.4)	9.5 (1.5–17.5)	8.1 (4.6–11.6)	10.0 (1.9–18.1)	8.3 (4.5–12.2)
**Number of drinking days**							
1 or 2 (n = 556)	23.7 (19.2–28.2)	34.9 (28.4–41.4)	10.2 (7.0–13.4)	5.0 (1.6–8.4)	4.2 (2.1–6.4)	10.9 (7.5–14.2)	11.1 (7.2–15.0)
3–9 (n = 412)	15.4 (10.2–20.6)	42.2 (36.9–47.6)	13.4 (9.4–17.4)	11.2 (6.4–15.9)	4.4 (1.9–6.8)	8.8 (5.7–12.0)	4.6 (2.1–7.1)
10–30 (n = 178)	14.2 (7.7–20.6)	32.7 (24.5–40.9)	11.3 (5.9–16.8)	13.5 (5.2–21.9)	6.5 (1.2–11.8)	11.1 (4.3–17.9)	10.7 (5.5–15.9)
**Largest number of drinks on any occasion**							
1–3 (n = 510)	21.7 (17.4–26.1)	36.6 (30.6–42.6)	9.5 (6.1–13.0)	6.3 (3.1–9.5)	4.3 (1.8–6.7)	10.2 (6.1–14.3)	11.3 (7.2–15.5)
4 or 5 (n = 188)	14.7 (8.0–21.4)	41.9 (33.8–50.0)	10.5 (4.9–16.0)	9.1 (5.2–13.0)	5.9 (2.2–9.6)	14.2 (6.9–21.4)	3.9 (0.7–7.0)
≥6 (n = 366)	12.3 (7.9–16.7)	41.0 (34.6–47.4)	13.9 (8.2–19.6)	10.9 (5.8–16.1)	5.1 (1.8–8.4)	8.9 (4.3–13.4)	7.8 (4.2–11.5)
**Mixed energy drinks and alcohol** (n = 404)	5.9 (2.1–9.7)	47.5 (42.2–52.7)	12.6 (8.2–17.1)	10.8 (6.4–15.1)	6.3 (3.3–9.3)	9.4 (5.4–13.4)	7.5 (4.5–10.5)

Abbreviation: CI, confidence interval.

a Survey question to assess usual types of alcohol was, “During the past 30 days, what type of liquor did you usually drink? (Select only one response)”.

b Nonbinge drinkers were defined as students who had at least 1 drink of alcohol on at least 1 day during the 30 days before the survey, but did not report binge drinking.

c Binge drinkers were defined as students who had 5 or more drinks of alcohol in a row within a couple of hours on at least 1 day during the 30 days before the survey.

d Other place was defined as bar or restaurant; while riding or driving in a car; at a park, beach, concert, or sporting event; or on school property.

Vodka was the most common liquor type among those who reported usually consuming liquor in the past 30 days (53.0%). Girls who reported usually consuming liquor had a higher prevalence of vodka consumption (60.3%) than boys (44.8%), but the difference between the 2 was not significant (data not shown).

## Discussion

To our knowledge, this is the first study to use the YTS to assess the types of alcohol consumed and the mixing of alcohol with energy drinks by high school students in a state. We found that more than half of the Michigan high school students who drank alcohol consumed liquor and that approximately half of the students who drank liquor usually consumed vodka. Liquor consumption was also associated with other dangerous drinking behaviors, including binge drinking and the mixing of alcohol and energy drinks. This finding is concerning because binge drinking increases the risk of alcohol-attributable harms, including sexual assault, and mixing of alcohol and energy drinks can increase binge drinking intensity, further increasing the risk of alcohol-attributable injuries and alcohol poisoning (15).

The finding that liquor is the usual type of alcohol consumed by Michigan high school students who drink is consistent with the findings of other studies (10–13). This is likely due to the fact that liquor has a higher alcohol concentration, is more portable and easily concealed than other alcoholic beverages, and can easily be mixed with other beverages, thereby making liquor more palatable to youth (16). Youth exposure to alcohol advertising in the United States increased by 71% from 2001 through 2009, largely because of an increase in liquor advertising on television (17), and youth exposure to alcohol advertising is associated with both the initiation of alcohol consumption by youth and the amount consumed per drinking occasion (18). The high prevalence of vodka consumption among high school students who drank liquor is also consistent with the reported increase in vodka sales in the United States and in Michigan (19,20), which has likely made vodka more accessible to youth, as well, particularly given that most high school students obtain the alcohol they consume from someone else (10).

The high prevalence of mixing liquor, such as vodka, with energy drinks, particularly among binge drinkers, is especially concerning because caffeine can mask some of the sensory cues that alert drinkers to their level of intoxication. This may lead youth to binge drink at higher intensity levels, increasing the risk of alcohol-attributable harms, including alcohol poisoning, motor vehicle crashes, and sexual assault (21). In fact, one study involving college students found that those who mixed energy drinks and alcohol were twice as likely to report being hurt or injured, riding in a car with an intoxicated driver, or requiring medical treatment than those who did not mix energy drinks and alcohol, even after adjusting for the amount of alcohol consumed (15).

Although liquor consumption was common among all Michigan high school students who were current drinkers, girls were somewhat more likely than boys to report consuming liquor. This is particularly concerning because women tend to achieve higher blood alcohol concentrations than men at the same consumption level, even taking into account differences in body size, food consumption, and other factors, thus increasing the risk of their experiencing alcohol-attributable harms, including unintended and alcohol-exposed pregnancies and adverse reproductive outcomes (22). These data highlight the need for additional and targeted efforts to prevent binge drinking among girls, including increased efforts to monitor and reduce youth exposure to alcohol marketing, particularly given that underage girls are overexposed to alcohol marketing relative to women to an even greater extent than underage boys are overexposed to alcohol marketing relative to men (23).

This study also demonstrated the potential usefulness of the YTS for assessing alcohol consumption among high school students. Although primarily focused on tobacco consumption among youth, the YTS has the flexibility to accommodate state-added questions on alcohol consumption, including questions on beverage-specific alcohol consumption, and is relatively inexpensive to administer. Furthermore, the collection of alcohol information in the YTS facilitates public health surveillance by providing a more specific assessment of the relationship between alcohol consumption and smoking by youth, including the potential impact of tobacco control measures and alcohol interventions on the drinking and smoking behaviors of high school students.

The results are subject to at least 7 limitations. First, this study assessed only the alcohol consumption patterns of Michigan students in 9th through 12th grade; therefore, these findings may not be generalizable to high school students in other states. Second, the YTS sample population was not large enough to assess significant differences between the demographic characteristics of liquor drinkers by type of liquor consumed (eg, vodka, rum, whiskey). Third, the YTS sample includes only students in public high schools, and although more than 90% of Michigan youth attend public schools, the results may not be representative of those who attend private or other nonpublic schools or students who do not attend school; previous research has demonstrated that students who attend alternative schools may have even higher rates of alcohol use than public school students (24). Fourth, all prevalence estimates are based on self-report, which are likely to underestimate alcohol consumption because of social desirability and recall biases (25). Fifth, about one-third of Michigan public high schools declined to participate in the YTS. The exclusion of the students in these schools from the survey may have affected the representativeness of the survey findings. Sixth, the data in this study were collected in 2011, and usual beverage consumption may have changed since then. However, liquor has consistently been reported to be the usual type of alcohol consumed by high school students in other studies (10–13), and it therefore seems unlikely this has changed since 2011. Finally, the definition of binge drinking that was used in the YTS (ie, 5 or more drinks within a couple of hours) was not sex-specific (26), and studies among women have shown that reducing the threshold for defining binge drinking from 5 drinks to 4 drinks increases the relative prevalence of binge drinking by more than one-third (27).

This study shows that current drinking, particularly binge drinking, by high school students in Michigan remains a serious public health problem and that high school students who drink are most likely to drink liquor, particularly vodka. Surveillance on beverage-specific alcohol consumption among youth can be useful for planning prevention strategies that target specific beverage types (eg, retail access to liquor) (9–11). Furthermore, because alcohol taxes are beverage-specific, knowing the type of alcoholic beverage usually consumed by youth can help inform alcohol tax policies in states and communities to prevent underage drinking (9–11). The Task Force on Community Preventive Services recommends several population-level, evidence-based strategies for preventing excessive alcohol use, including underage drinking, that can help reduce beverage-specific alcohol consumption by youth. These include increasing alcohol excise taxes, regulating alcohol outlet density, establishing commercial host liability, enhanced enforcement of the age 21 minimum legal drinking-age (eg, through compliance checks in which minors or youthful-looking adults attempt to purchase alcohol from retail establishments), and avoiding privatization of alcohol sales (28). Previous research has demonstrated that teenagers and young adults are especially responsive to increases in the price of alcohol (29). Improved compliance with the voluntary industry threshold for the placement of alcohol advertising in television programs and in other media venues (ie, 28.4% youth audience composition), particularly advertising that is placed on cable nonsports television programs, could also help reduce the risk of underage drinking (30). Finally, states can routinely assess beverage-specific alcohol consumption using the YTS and use these data to help guide efforts to reduce youth exposure to alcohol marketing. For example, the Michigan Department of Health and Human Services has worked closely with state and local coalitions to collect and report information on excessive alcohol use and related harms and to develop a statewide plan to reduce underage drinking.

## References

[1] Stahre M , Roeber J , Kanny D , Brewer RD , Zhang X . Contribution of excessive alcohol consumption to deaths and years of potential life lost in the United States. Prev Chronic Dis 2014;11:E109. 10.5888/pcd11.130293 24967831 PMC4075492

[2] Bouchery EE , Harwood HJ , Sacks JJ , Simon CJ , Brewer RD . Economic costs of excessive alcohol consumption in the US, 2006. Am J Prev Med 2011;41(5):516–24. 10.1016/j.amepre.2011.06.045 22011424

[3] Office of Juvenile Justice and Delinquency Prevention. Drinking in America: myths, realities, and prevention policy. Washington (DC): US Department of Justice, Office of Justice Programs, Office of Juvenile Justice and Delinquency Prevention, 2005.

[4] US Department of Health and Human Services. The Surgeon General’s Call to Action To Prevent and Reduce Underage Drinking. Washington (DC): US Department of Health and Human Services, Office of the Surgeon General; 2007. 20669519

[5] Bonnie RJ , O’Connell ME , editors. Reducing underage drinking: a collective responsibility. Washington (DC): The National Academies Press; 2004. http://www.nap.edu/openbook.php?isbn=0309089352. Accessed August 21, 2015. 20669473

[6] Miller JW , Naimi TS , Brewer RD , Jones SE . Binge drinking and associated health risk behaviors among high school students. Pediatrics 2007;119(1):76–85. 10.1542/peds.2006-1517 17200273

[7] Nelson DE , Naimi TS , Brewer RD , Nelson HA . State alcohol-use estimates among youth and adults, 1993–2005. Am J Prev Med 2009;36(3):218–24. 10.1016/j.amepre.2008.10.018 19215847

[8] Wagenaar AC , Toomey TL , Murray DM , Short BJ , Wolfson M , Jones-Webb R . Sources of alcohol for underage drinkers. J Stud Alcohol 1996;57(3):325–33. 10.15288/jsa.1996.57.325 8709591

[9] Centers for Disease Control and Prevention. Types of alcoholic beverages usually consumed by students in 9th–12th grades — four states, 2005. MMWR Morb Mortal Wkly Rep 2007;56(29):737–40. 17657207

[10] Cremeens JL , Miller JW , Nelson DE , Brewer RD . Assessment of source and type of alcohol consumed by high school students: analyses from four States. J Addict Med 2009;3(4):204–10. 10.1097/ADM.0b013e31818fcc2c 21769017

[11] Siegel MB , Naimi TS , Cremeens JL , Nelson DE . Alcoholic beverage preferences and associated drinking patterns and risk behaviors among high school youth. Am J Prev Med 2011;40(4):419–26. 10.1016/j.amepre.2010.12.011 21406275

[12] Siegel M , DeJong W , Naimi TS , Heeren T , Rosenbloom DL , Ross C , Alcohol brand preferences of underage youth: results from a pilot survey among a national sample. Subst Abus 2011;32(4):191–201. 10.1080/08897077.2011.601250 22014249 PMC3202336

[13] Tanski SE , McClure AC , Jernigan DH , Sargent JD . Alcohol brand preference and binge drinking among adolescents. Arch Pediatr Adolesc Med 2011;165(7):675–6. 10.1001/archpediatrics.2011.113 21727284 PMC3312779

[14] Thombs DL , O’Mara RJ , Tsukamoto M , Rossheim ME , Weiler RM , Merves ML , Event-level analyses of energy drink consumption and alcohol intoxication in bar patrons. Addict Behav 2010;35(4):325–30. 10.1016/j.addbeh.2009.11.004 19954894

[15] O’Brien MC , McCoy TP , Rhodes SD , Wagoner A , Wolfson M . Caffeinated cocktails: energy drink consumption, high-risk drinking, and alcohol-related consequences among college students. Acad Emerg Med 2008;15(5):453–60. 10.1111/j.1553-2712.2008.00085.x 18439201

[16] Centers for Disease Control and Prevention (CDC). Alcohol use among high school students — Georgia, 2007. MMWR Morb Mortal Wkly Rep 2009;58(32):885–90. 19696717

[17] Center on Alcohol Marketing and Youth. Youth exposure to alcohol advertising on television, 2001–2009. Washington, DC, 2012. http://www.camy.org/research/Youth_Exposure_to_Alcohol_Ads_on_TV_Growing_Faster_Than_Adults/_includes/TVReport01-09_Revised_7-12.pdf. Accessed August 28, 2015.

[18] Anderson P , de Bruijn A , Angus K , Gordon R , Hastings G . Impact of alcohol advertising and media exposure on adolescent alcohol use: a systematic review of longitudinal studies. Alcohol Alcohol 2009;44(3):229–43. 10.1093/alcalc/agn115 19144976

[19] Distilled Spirits Council of the United States. Vodka: the spirit of the industry. http://www.discus.org/assets/1/7/Vodka2010.pdf. Accessed August 28, 2015.

[20] Michigan Liquor Control Commission. Annual financial report; 2011. http://www.michigan.gov/documents/lara/statbook2011_new_397864_7.pdf. Accessed August 28, 2015.

[21] Ferreira SE , de Mello MT , Pompéia S , de Souza-Formigoni ML . Effects of energy drink ingestion on alcohol intoxication. Alcohol Clin Exp Res 2006;30(4):598–605. 10.1111/j.1530-0277.2006.00070.x 16573577

[22] Naimi TS , Lipscomb LE , Brewer RD , Gilbert BC . Binge drinking in the preconception period and the risk of unintended pregnancy: implications for women and their children. Pediatrics 2003;111(5 Pt 2):1136–41. 12728126

[23] Jernigan DH , Ostroff J , Ross C , O’Hara JA 3d . Sex differences in adolescent exposure to alcohol advertising in magazines. Arch Pediatr Adolesc Med 2004;158(7):629–34. 10.1001/archpedi.158.7.629 15237061

[24] Grunbaum JA , Lowry R , Kann L . Prevalence of health-related behaviors among alternative high school students as compared with students attending regular high schools. J Adolesc Health 2001;29(5):337–43. 10.1016/S1054-139X(01)00304-4 11691595

[25] Davis CG , Thake J , Vilhena N . Social desirability biases in self-reported alcohol consumption and harms. Addict Behav 2010;35(4):302–11. 10.1016/j.addbeh.2009.11.001 19932936

[26] National Institute on Alcohol Abuse and Alcoholism. NIAAA Council approves binge drinking defınition. NIAAA Newsletter 2004;(3):3. Alcohol Abuse and Alcoholism. http://pubs.niaaa.nih.gov/publications/Newsletter/winter2004/Newsletter_Number3.pdf. Accessed August 28, 2015.

[27] Chavez PR , Nelson DE , Naimi TS , Brewer RD . Impact of a new gender-specific definition for binge drinking on prevalence estimates for women. Am J Prev Med 2011;40(4):468–71. 10.1016/j.amepre.2010.12.008 21406282 PMC3090660

[28] Task Force on Community Preventive Services. Preventing excessive alcohol consumption. In: The guide to community preventive services. New York (NY): Oxford University Press; 2005. http://www.thecommunityguide.org/alcohol/index.html. Accessed on April 28, 2014.

[29] Xu X , Chaloupka FJ . The effects of prices on alcohol use and its consequences. Alcohol Res Health 2011;34(2):236–45. 22330223 PMC3860576

[30] Centers for Disease Control and Prevention. Youth exposure to alcohol advertising on television — 25 markets, United States, 2010. MMWR Morb Mortal Wkly Rep 2013;62(44):877–80. 24196664 PMC4585591

